# Planetary health diet: dissecting the link between diet, mortality risk and heart age from a 16-year follow-up of the Guangzhou Biobank Cohort Study

**DOI:** 10.1007/s00394-026-04002-x

**Published:** 2026-05-26

**Authors:** Ting Yu Lu, Jiao Wang, Ying Yue Huang, Wen Bo Tian, Ya Li Jin, Tai Hing Lam, Wei Sen Zhang, Lin Xu

**Affiliations:** 1 School of Public Health, Sun Yat-sen University, 74 Zhongshan 2nd Road, Guangzhou, 510080 Guangdong Province China; 2 Guangzhou Twelfth People’s Hospital, Guangzhou, 510620 China; 3 School of Public Health, The University of Hong Kong, Hong Kong, China; 4 Department of Applied Health Sciences, University of Birmingham, Birmingham, B15 2TT UK; 5 Greater Bay Area Public Health Research Collaboration, Guangdong, China

**Keywords:** Planetary health diet, Mortality, Heart age, Cohort study, Mediation analysis

## Abstract

**Purpose:**

To examine the associations of planetary health diet (PHD) with all-cause and cause specific mortality, alongside heart age based on the Guangzhou Biobank Cohort Study (GBCS) and conduct mediation analysis.

**Methods:**

Participants were recruited from the GBCS and were aged ≥ 50 years. Dietary information was collected using a validated Food Frequency Questionnaire. Participants were assigned PHD scores between 0 (no adherence to PHD) and 140 (complete adherence to PHD). Primary outcomes were all-cause, cardiovascular disease (CVD) and cancer mortality. Causes of death were identified through death registry. Secondary outcome, heart age, was calculated using sex-specific 10-year CVD risk prediction models previously developed and validated in the GBCS. Cox proportional hazards regression and linear regression were used to analyze the associations of PHD scores with mortality and heart age. Mediation analyses were conducted using the difference method implemented by the “mediate” SAS macro.

**Results:**

Of 25,550 participants aged 50+ years, during 417,590 person-years of follow-up, higher PHD scores was linearly associated with lower all-cause and CVD but not cancer mortality (hazard ratio (HR) (95% confidence interval (CI)) per 10-point increment: 0.94 (0.92–0.97), 0.92 (0.89–0.95) and 0.97 (0.93–1.01)). The association with all-cause mortality was mediated by white blood cell count (WBC), waist-to-hip ratio and waist-to-hip-to-height ratio (mediation proportion (95% CI): 6.2% (3.2–11.7%), 2.6% (0.9–7.2%) and 5.4% (2.8–9.9%)), whereas the association with CVD mortality was mediated by WBC and waist-to-hip-to-height ratio (7.9% (4.1–14.9%) and 7.4% (3.0–17.0%)). A negative association between PHD scores and heart age was observed in women but not in men (β (95% CI) per 10-point increment: − 0.13 (− 0.24, -0.01) and 0.05 (− 0.15, 0.25) years, P_interaction_ < 0.001).

**Conclusion:**

Higher adherence to PHD was linearly associated with lower all-cause and CVD but not cancer mortality in Chinese aged 50+ years, and with lower heart age in women only. Our findings advocate for PHD in middle-aged to older Chinese, particularly women to improve cardiovascular health.

**Supplementary Information:**

The online version contains supplementary material available at 10.1007/s00394-026-04002-x.

## Introduction

Diet is not only a modifiable risk factor for health-related outcomes, but also an important link between human health and environmental sustainability. In 2019, the EAT-Lancet Commission proposed the planetary health diet (PHD) as a healthy and environmentally sustainable dietary pattern for all food cultures [1], emphasizing plant-based foods and limited animal foods, sugar and saturated fats. Since then, 15 PHD scores have been developed [2–16], primarily based on Western populations [2–12, 14–16], with only one derived from Asian population (Singapore) [13]. Given the substantial differences in dietary customs between Western and Asian populations, evidence from Western populations cannot be directly generalized to other regions.

PHD has been reported to be associated with lower risks of all-cause or cardiovascular disease (CVD) mortality [2, 8, 9, 13, 15, 17], which may be partly attributable to its emphasis on plant-based foods intake and low amounts of red meat, sugar or saturated fats, as well as its potential effects on metabolic health [18], inflammation [19, 20] and obesity indicators [21]. However, the association between PHD and cancer mortality was inconsistent, with some studies showing a negative association [8, 9], while others reported non-significant results [13, 15]. Furthermore, previous studies have shown the potential impact of PHD on certain CVD risk factors [18] and CVD incidence [22], but limited research has examined its association with overall predicted CVD risk [18]. Since overall predicted cardiovascular health indicators, such as heart age, can support primary prevention strategies by facilitating communication of absolute CVD risk and early prevention of subclinical CVD [23], exploring the association between adherence to the PHD and these indicators is of public health importance.

Moreover, animal-based protein has been reported to provide essential amino acids for preventing muscle loss in the elderly, which may contribute to improved health outcomes, including reduced mortality risk [24].Given potential concerns regarding the nutritional adequacy of plant-based diets in older populations, it remains unclear whether the PHD confers similar benefits in this age group. Examining the health effects of PHD (e.g., mortality risks) in older adults may provide evidence to address these concerns.

Hence, using data from the Guangzhou Biobank Cohort Study (GBCS), we aimed to assess the associations of PHD with all-cause, CVD and cancer mortality in middle-aged to older Chinese. We also conducted mediation analyses to explore the role of metabolic health, inflammation and obesity indicators in these associations. Furthermore, to enhance communication of CVD risk to the public, we calculated heart age based on a 10-year CVD risk prediction model and examined the association of PHD with heart age.

## Materials and methods

### Study sample

All participants in the GBCS were recruited at baseline across three phases (September 2003 to January 2008), including phase 1 (2003–2004), phase 2 (2005–2006), and phase 3 (2006–2008). Details of GBCS have been reported previously [25]. Briefly, GBCS is a 3-way collaboration among the Guangzhou Twelfth People’s Hospital and the Universities of Hong Kong, China, and Birmingham, UK. Participants were recruited from Guangzhou Health and Happiness Association for the Respectable Elders, a community social and welfare organization. Membership was open to Guangzhou permanent residents aged 50 years or older for a nominal fee of 4 CNY (≈50 US cents) per month. The baseline examination included a face-to-face, computer-assisted interview by trained nurses to collect information on demographic characteristics, lifestyle factors, family and personal medical history. Anthropometric parameters, blood pressure, fasting plasma glucose, lipids and inflammatory markers were measured. The reliability of the questionnaire was tested by re-interviewing 200 participants, randomly selected from the initial cohort. The results indicated good reproducibility, with high kappa values for categorical variables (e.g., smoking (0.96 and 0.88 for the two questions on smoking status), education (0.90), occupation (0.80)) and moderate to high intraclass correlation coefficients for continuous variables (e.g., age at menarche (0.92) and age at first pregnancy (0.50)) [25]. The Guangzhou Medical Ethics Committee of the Chinese Medical Association approved the study (protocol code: GZYL-20030210), and all participants gave written, informed consent before participation. Among 30,430 GBCS participants aged 50 years or older, we excluded those with implausible energy intake, defined as < 800 or > 4200 kcal/day for men, and < 600 or > 3500 kcal/day for women [26], those with missing information on PHD scores (*N* = 1,426), CVD or cancer at baseline (*N* = 3,106), and those lost to follow-up with unknown vital status (*N* = 348). This left 25,550 participants (8,938 from phase 1, 8,752 from phase 2, and 7,680 from phase 3) for the main analysis (Supplementary Figs. 1 and 2).

### Exposure

The exposure variable was PHD scores. Information on the amounts and frequencies of 303 food items consumption was obtained from a validated Food Frequency Questionnaire (FFQ) in the first two phases at baseline, which was simplified in phase 3 to reduce respondent burden. Each food item’s consumption was calculated by multiplying the frequency by the amount, and total energy intake was calculated by multiplying the daily food intake and energy of each type of food based on the sixth edition of the Chinese Food Composition Tables [27]. To harmonize dietary data across phases, individual food items from the comprehensive FFQ were categorized into the corresponding food groups in the simplified FFQ. In phase 3, the median energy value of all individual food items within each food category was used as the energy estimate for that group. Daily energy intake was calculated as the sum of energy intake across food groups, obtained by multiplying the estimated energy value by the intake level of each food group. The mean (standard deviation (SD)) daily energy intake level in the first two phases was 1825.89 (511.47) kcal/day, which was comparable to that calculated from the simplified FFQ in phase 3 (1898.86 (494.29) kcal/day). Details of the FFQ in our cohort have been reported elsewhere [28]. All daily food intakes were standardized to an energy intake of 2500 kcal/day using the following formula: standardized food intake level = actual food intake level × (2500 kcal/day ÷ actual daily energy intake). Subsequently, following the EAT-Lancet report and previous studies [5, 29], we categorized all the food items into 14 food groups and calculated daily intake for each food group (g/day). The 14 food groups were further divided into three major categories, i.e., emphasized, optional and limited foods, each with specific scoring criteria. Details of scoring criteria and representative food items within each food category are shown in Supplementary Tables 1 and 2. Participants were assigned continuous scores based on intake levels ranging from 0 to 10 points per food group, which were then summed to obtain the total PHD scores, ranging from 0 (no adherence to PHD) to 140 (complete adherence to PHD).

### Outcomes

The primary outcomes were all-cause, CVD and cancer mortality. Information on causes of death up to November 2023 was primarily obtained via record linkage with the Death Registry of the Guangzhou Center for Disease Control and Prevention (GCDC). Causes of death were coded by trained medical staff in each hospital according to the 10th revision of the International Classification of Diseases (ICD-10), including CVD (I00-I25, I28-I99) and cancer (C00-C97). For deaths not certified by medical institutions, which might have coding quality issue, the causes of death were verified by GCDC as part of its quality assurance program by cross-checking past medical histories and conducting verbal autopsies [30].

To enhance CVD risk communication with the public, we calculated heart age as a secondary outcome. Heart age was defined as the age of an individual with the same predicted CVD risk but with all other risk factors in normal ranges [23]. In this study, heart age was calculated based on the sex-specific 10-year CVD risk prediction models previously developed in the GBCS, which showed good discrimination in both women (c-statistic 0.72, 95% CI 0.71–0.73) and men (c-statistic 0.68, 95% CI 0.67–0.70) [31]. First, we calculated the predicted 10-year CVD risk using simple predictors including age, systolic blood pressure (SBP), use of antihypertensive medication, smoking and diabetes. Second, heart age was determined as the age of someone with the same predicted CVD risk but with all predictors at normal levels, i.e., SBP of 120 mmHg, non-smoker, not with diabetes nor antihypertensive medication use. A lower heart age indicated better cardiovascular health.

### Potential confounders and mediators

As both dietary intake and mortality risk may be influenced by sex, age, social-economic position (education, occupation and family income) [32], personal lifestyles (smoking status, alcohol use and physical activity) [33], self-rated or objective health status [34] and BMI [35], these factors were considered potential confounders and included in the analyses. Self-rated health was assessed by asking participants to rate their health status compared to others of the same age. Poor objective health status was defined as the presence of any of the following conditions: (1) regular use of medication for chronic diseases in the past 30 days, (2) any hospital admission during the past 6 months, (3) self-reported CVD history, or (4) self-reported cancer history. Besides, since we harmonized nutritional data from the first two phases and phase 3 in GBCS, we additionally adjusted for recruitment phase in the main analyses.

To further explore the potential mechanisms, we also used baseline data to comprehensively assess the cross-sectional associations of PHD scores with metabolic health, inflammation and obesity indicators, including fasting plasma glucose, blood pressure (SBP and diastolic blood pressure (DBP)), lipids (total cholesterol, high-density lipoprotein-cholesterol (HDL-C), low-density lipoprotein-cholesterol (LDL-C) and triglycerides), white blood cell count (WBC), hypersensitive C-reactive protein (hsCRP), albumin, body mass index (BMI), waist circumference, waist-to-hip ratio, waist-to-height ratio, waist-to-hip-to-height ratio and body roundness index. Subsequently, we selected the risk factors showing significant associations with PHD scores (i.e., all groups showed results in the same direction, among them any group showed significant results, and P_trend_ < 0.05) to conduct mediation analyses for mortality risks.

### Statistical analyses

We categorized PHD scores of the study sample into quintiles, with the lowest quintile serving as the reference. One-way analysis of variance (ANOVA) and chi-square test were used to compare baseline continuous and categorical variables across PHD score groups, respectively. Schoenfeld residuals were used to test the proportional hazard assumption. Since no evidence of violation for the proportional hazard assumption was found (P from 0.06 to 0.75), Cox proportional hazards regression was used to examine the associations of PHD scores with all-cause, CVD and cancer mortality, yielding hazard ratios (HRs) and 95% confidence intervals (CIs). Three models were conducted, including a crude model, model 1, adjusting for sex, age, education, occupation, family income, smoking status, alcohol use, physical activity, self-rated health, objective health status and recruitment phase, and model 2, additionally adjusting for BMI. We used restricted cubic spline (RCS) plots with four knots placed at the 20th, 40th, 60th, and 80th percentiles of the PHD score distribution, with a reference value of 60 points (approximately corresponding to the mean and median PHD score in this study), to flexibly model the associations of PHD scores with mortality risks, accompanied by Wald-type test to evaluate potential nonlinearity. In participants without CVD at baseline, linear regression was used to assess the association between PHD scores and heart age, yielding βs and 95% CIs. Before conducting the mediation analyses, cross-sectional associations of PHD scores with metabolic health, inflammation and obesity indicators at baseline were examined, adjusting for sex, age, education, occupation, family income, smoking status, alcohol use, physical activity, self-rated health, objective health status, recruitment phase and BMI (except when obesity indicators were the outcomes). For biomarkers showing significant associations with PHD scores, the difference method [36], implemented using the publicly available “mediate” SAS macro, which quantifies the difference in estimates obtained from separate exposure-outcome models with and without the mediator (https://ysph.yale.edu/cmips/research/software/mediate_340185_284_47911_v2.pdf), was used to estimate the mediation proportions for the associations between PHD scores and mortality risks.

Several sensitivity analyses were conducted to examine the robustness of our findings. First, we excluded deaths occurring within the first two years of follow-up to reduce reverse causality. Second, we examined the interactions of PHD scores with some potential moderators (i.e., sex [37], age [38] and BMI [39]) on mortality and heart age by comparing fitness of the models before and after adding the interaction terms using log-likelihood ratio test, and also conducted subgroup analyses stratifying by them. Moreover, since we harmonized data from all the three phases in the main analyses, we also used data in the first two phases, with more precise dietary information, to repeat the main analyses in order to examine the robustness of our results. Stata/SE 16.0 (Stata Corp LP) and SAS 9.4 were used for data analyses. All tests were two-sided, and *P* < 0.05 was considered statistically significant.

## Results

### Baseline characteristics

Of 25,550 participants, over 417,590 person-years of follow-up (mean 16.3 years), 6,607 deaths occurred, including 2,562 CVD and 2,024 cancer deaths. The mean (SD) PHD score among the 25,550 participants was 62.11 (11.57) points. Participants in the highest quintile of PHD scores had higher proportion of women, non-manual workers, never smokers and current drinkers (all *P* < 0.001) (Table 1). They were also younger, more physically active, had higher socioeconomic or educational levels, higher BMI and better self-rated health (all *P* < 0.001), but had worse objective health status (*P* = 0.023).

**Table 1:** Baseline characteristics by planetary health diet score quintiles on 25,550 Guangzhou Biobank Cohort Study (GBCS) participants in 2003–2008

	Overall	Planetary health diet score (range: 0–140)					*P* value
		Quintile 1 (lowest adherence)	Quintile 2	Quintile 3	Quintile 4	Quintile 5 (highest adherence)	
Number of participants, *N* (%)	25,550 (100.00)	5143 (20.13)	5128 (20.07)	5110 (20.00)	5078 (19.87)	5091 (19.93)	–
PHD score, mean (SD)	62.11 (11.57)	45.97 (5.20)	55.94 (1.99)	62.14 (1.68)	68.39 (1.95)	78.36 (5.40)	< 0.001
Sex, %							< 0.001
Men	27.92	32.32	29.49	29.04	25.82	22.88	
Women	72.08	67.68	70.51	70.96	74.18	77.12	
Age, years, mean (SD)	61.80 (7.06)	62.76 (6.97)	62.07 (7.05)	61.78 (7.01)	61.42 (7.08)	60.96 (7.05)	< 0.001
Family income, CNY/year, %							< 0.001
< 10,000	5.69	9.45	6.25	4.82	4.26	3.64	
10,000–29,999	32.25	35.20	32.75	33.27	31.33	28.64	
30,000–49,999	21.26	16.81	20.04	21.45	21.84	26.22	
≥ 50,000	16.88	11.94	14.98	16.65	19.37	21.52	
Don’t know	23.93	26.59	25.99	23.82	23.20	19.99	
Education, %							< 0.001
Primary or below	42.71	55.77	47.27	41.81	37.40	31.12	
Secondary	48.60	38.44	44.85	49.44	53.23	57.19	
College or above	8.69	5.80	7.88	8.75	9.37	11.69	
Occupation, %							< 0.001
Manual	61.68	69.22	64.26	61.56	58.59	54.62	
Non-manual	23.33	17.03	20.66	23.39	26.10	29.57	
Others	15.00	13.75	15.08	15.05	15.31	15.81	
Smoking status, %							< 0.001
Never	80.84	74.71	79.12	80.52	82.92	86.98	
Former	8.96	10.65	9.60	9.43	8.14	6.96	
Current	10.20	14.64	11.28	10.04	8.94	6.06	
Alcohol use, %							< 0.001
Never	70.58	76.89	73.40	69.57	68.44	64.15	
Former	3.58	3.32	3.49	3.84	3.69	3.56	
Current	25.84	19.79	23.12	26.59	27.87	32.28	
Physical activity, %							< 0.001
Inactive	7.94	13.07	8.87	6.65	5.85	5.21	
Moderate	41.15	46.32	43.62	41.21	38.64	35.87	
Active	50.91	40.62	47.50	52.13	55.51	58.93	
Self-rated health, %							< 0.001
Good	84.02	82.66	82.80	84.75	84.57	85.38	
Poor	15.98	17.34	17.20	15.25	15.43	14.62	
Objective health status							0.023
Good	86.27	87.36	86.62	86.26	85.88	85.19	
Poor	13.73	12.64	13.38	13.74	14.12	14.81	
BMI, kg/m^2^, mean (SD)	23.71 (3.28)	23.54 (3.33)	23.67 (3.31)	23.69 (3.28)	23.75 (3.25)	23.90 (3.23)	< 0.001

N, number; SD, standard deviation; PHD, planetary health diet; BMI, body mass index; CNY, Chinese Yuan

### PHD scores and mortality

**Fig. 1:**
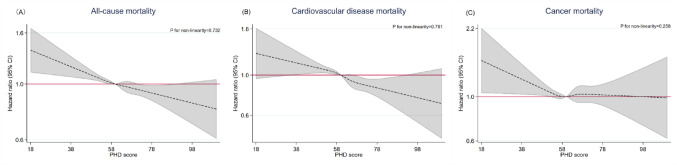
Restricted cubic spline plots for the associations of baseline planetary health diet scores with all-cause, cardiovascular disease and cancer mortality on 25,550 Guangzhou Biobank Cohort Study (GBCS) participants in 2003–2008 and followed up till November 2023. **A** all-cause mortality; **B** cardiovascular disease mortality; **C** cancer mortality. The HRs and 95% CIs above were adjusted for sex, age, education, occupation, family income, smoking status, alcohol use, physical activity, self-rated health, objective health status, recruitment phase and body mass index. HR, hazard ratio; CI, confidence interval; PHD, planetary health diet

Table 2 shows that in the fully adjusted model 2, compared with the lowest quintile, higher PHD scores was significantly associated with lower risk of all-cause mortality. The HR (95% CI) was 0.89 (0.83–0.96), 0.93 (0.86–0.99), 0.85 (0.79–0.92) and 0.82 (0.75–0.89) from Quintile 2 to Quintile 5, respectively (P_trend_ < 0.001). Similarly, for CVD mortality, the HRs (95% CIs) from Quintile 2 to Quintile 5 were 0.95 (0.85–1.07), 0.89 (0.78–1.002), 0.81 (0.72–0.92) and 0.76 (0.67–0.87), respectively (P_trend_ < 0.001). For cancer mortality, the corresponding HRs (95% CIs) from Quintile 2 to Quintile 5 were 0.82 (0.71–0.95), 0.99 (0.86–1.13), 0.86 (0.74–0.99), and 0.89 (0.77–1.03), respectively (P_trend_ = 0.209). An increase in PHD scores (per 10 points) was significantly associated with lower risks of all-cause and CVD mortality, but not cancer mortality (HR (95% CI) was 0.94 (0.92–0.97), 0.92 (0.89–0.95) and 0.97 (0.93–1.01), respectively). RCS plots further show the linear relationships of PHD scores with all-cause and CVD mortality, but not cancer mortality. For cancer mortality, no overall linear association was observed; however, below the reference value of 60 points, higher PHD scores were associated with a lower risk of cancer mortality (Fig. 1).

**Table 2:** Associations of baseline planetary health diet scores with all-cause, cardiovascular disease and cancer mortality on 25,550 Guangzhou Biobank Cohort Study (GBCS) participants in 2003–2008 and followed up till November 2023

	Planetary health diet score (range: 0–140), HR (95% CI)						P for trend
	Quintile 1 (lowest adherence)	Quintile 2	Quintile 3	Quintile 4	Quintile 5 (highest adherence)	Per 10-point increment	
Person-years	83,324	83,561	83,212	83,584	83,911	417,590	–
All-cause mortality							
No. of deaths	1664	1383	1341	1177	1042	6607	–
Mortality rate, per 10,000 person-years	199.7	165.5	161.2	140.8	124.2	158.2	–
Crude model	1.00	0.83 (0.78, 0.89)***	0.81 (0.75, 0.87)***	0.71 (0.66, 0.76)***	0.63 (0.58, 0.68)***	0.87 (0.85, 0.89)^***^	< 0.001
Model 1	1.00	0.90 (0.83, 0.97)**	0.93 (0.86, 1.0004)	0.85 (0.79, 0.92)***	0.82 (0.75, 0.89)***	0.94 (0.92, 0.97)^***^	< 0.001
Model 2	1.00	0.89 (0.83, 0.96)**	0.93 (0.86, 0.99) ^*^	0.85 (0.79, 0.92)***	0.82 (0.75, 0.89)***	0.94 (0.92, 0.97)***	< 0.001
Cardiovascular disease mortality							
No. of deaths	663	565	509	447	378	2562	–
Mortality rate, per 10,000 person-years	79.6	67.6	61.2	53.5	45.0	61.4	–
Crude model	1.00	0.86 (0.76, 0.96)**	0.77 (0.69, 0.87)***	0.68 (0.60, 0.76)***	0.58 (0.51, 0.66)***	0.85 (0.82, 0.88)***	< 0.001
Model 1	1.00	0.95 (0.85, 1.07)	0.89 (0.79, 1.004)	0.82 (0.72, 0.93)**	0.77 (0.67, 0.88)***	0.92 (0.89, 0.95)***	< 0.001
Model 2	1.00	0.95 (0.85, 1.07)	0.89 (0.78, 1.002)	0.81 (0.72, 0.92)**	0.76 (0.67, 0.87)***	0.92 (0.89, 0.95)***	< 0.001
Cancer mortality							
No. of deaths	487	387	438	363	349	2024	
Mortality rate, per 10,000 person-years	58.4	46.3	52.6	43.4	41.6	48.5	
Crude model	1.00	0.79 (0.69, 0.90)**	0.90 (0.79, 1.02)	0.74 (0.65, 0.85)***	0.71 (0.62, 0.81)***	0.90 (0.87, 0.94)***	< 0.001
Model 1	1.00	0.83 (0.72, 0.96) ^*^	0.99 (0.86, 1.13)	0.86 (0.75, 0.99) ^*^	0.89 (0.77, 1.03)	0.97 (0.93, 1.01)	0.204
Model 2	1.00	0.82 (0.71, 0.95)**	0.99 (0.86, 1.13)	0.86 (0.74, 0.99) ^*^	0.89 (0.77, 1.03)	0.97 (0.93, 1.01)	0.209

HR, hazard ratio; CI, confidence interval

Model 1: adjusted for sex, age, education, occupation, family income, smoking status, alcohol use, physical activity, self-rated health, objective health status and recruitment phase

Model 2: additionally adjusted for body mass index

^*^P < 0.05, ^**^P < 0.01, ^***^P < 0.001

### Cross-sectional and mediation analyses

**Fig. 2:**
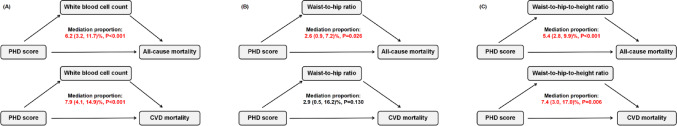
Potential mediations for the associations of baseline planetary health diet scores with all-cause or cardiovascular disease mortality in Guangzhou Biobank Cohort Study (GBCS) participants in 2003–2008 and followed up till November 2023. **A** White blood cell count; **B** waist-to-hip ratio; **C** waist-to-hip-to-height ratio. The mediation analyses were adjusted for sex and age. PHD, planetary health diet; CI, confidence interval

In cross-sectional analyses of baseline data, fully adjusted model 1 showed that higher PHD scores (Quintile 4 or 5) was significantly associated with lower levels of triglycerides, WBC, waist-to-hip ratio and waist-to-hip-to-height ratio, but higher levels of BMI (all P_trend_ < 0.05, Supplementary Table 3. Compared with the lowest quintile, participants in Quintile 2 had higher SBP (β (95% CI): 0.83 (0.05, 1.61) mmHg), while those in Quintile 4 had significantly lower DBP (β (95% CI): − 0.43 (− 0.85, − 0.02) mmHg). In mediation analyses, the association of higher PHD scores with lower all-cause mortality was mediated by WBC, waist-to-hip ratio and waist-to-hip-to-height ratio, with mediation proportion (95% CI) of 6.2% (3.2–11.7%), 2.6% (0.9–7.2%) and 5.4% (2.8–9.9%), respectively (all *P* < 0.05). The negative association between PHD scores and CVD mortality was mediated by WBC and waist-to-hip-to-height ratio, with mediation proportion (95% CI) of 7.9 (4.1–14.9%) and 7.4 (3.0–17.0%), respectively (*P* < 0.01), but not by waist-to-hip ratio. No significant mediations were found for triglycerides or BMI in the associations between PHD scores and all-cause or CVD mortality (Fig. 2 and Supplementary Table 4).

### PHD scores and heart age

Table 3 shows that, in the fully adjusted model 2, compared with the lowest quintile, higher PHD scores was non-significantly associated with lower heart age. When considering the PHD scores a continuous variable, there was a marginally non-significant association between an increase in PHD scores (per 10 points) and lower heart age (β (95% CI): − 0.08 (− 0.18, 0.02) years). As a significant interaction was found between PHD scores and sex on heart age (P_interaction_ < 0.001), stratification analysis by sex was performed. The significant association between higher PHD scores and lower heart age was evident in women (β (95% CI) per 10-point increment: − 0.13 (− 0.24, -0.01) years, P_trend_ < 0.05), but not in men (β (95% CI) per 10-point increment: 0.05 (− 0.15, 0.25) years, P_trend_ = 0.744) (Table 3).

**Table 3:** Association of planetary health diet scores with heart age on 26,314 Guangzhou Biobank Cohort Study (GBCS) participants without baseline cardiovascular disease in 2003–2008 and stratified by sex

	Heart age, mean (SD), years	Heart age, years, β (95% CI)		
		Crude model	Model 1	Model 2
All participants				
Quintile 1 (lowest adherence)	66.98 (10.23)	0.00	0.00	0.00
Quintile 2	66.13 (10.33)	− 0.85 (− 1.24, − 0.46)***	0.001 (− 0.34, 0.34)	− 0.01 (− 0.35, 0.33)
Quintile 3	65.65 (10.20)	− 1.33 (− 1.72, − 0.94)***	− 0.07 (− 0.41, 0.28)	− 0.09 (− 0.44, 0.25)
Quintile 4	64.90 (10.12)	− 2.08 (− 2.47, − 1.69)***	− 0.27 (− 0.63, 0.08)	− 0.31 (− 0.66, 0.04)
Quintile 5 (highest adherence)	64.38 (10.19)	− 2.59 (− 2.98, − 2.20)***	− 0.10 (− 0.46, 0.26)	− 0.17 (− 0.53, 0.19)
Per 10-point increment	65.61 (10.25)	− 0.81 (− 0.91, − 0.70)***	− 0.06 (− 0.16, 0.04)	− 0.08 (− 0.18, 0.02)
* P* for trend	–	< 0.001	0.247	0.114
Men				
Quintile 1 (lowest adherence)	70.05 (9.92)	0.00	0.00	0.00
Quintile 2	70.63 (10.25)	0.58 (− 0.11, 1.27)	0.75 (0.10, 1.40)^*^	0.70 (0.06, 1.35)^*^
Quintile 3	70.00 (10.12)	− 0.04 (− 0.74, 0.65)	0.16 (− 0.50, 0.82)	0.10 (− 0.56, 0.76)
Quintile 4	69.65 (9.89)	− 0.40 (− 1.11, 0.32)	− 0.11 (− 0.80, 0.58)	− 0.20 (− 0.88, 0.49)
Quintile 5 (highest adherence)	70.39 (10.05)	0.35 (− 0.39, 1.09)	0.77 (0.05, 1.50)^*^	0.62 (− 0.10, 1.34)
Per 10-point increment	70.15 (10.03)	− 0.01 (− 0.21, 0.20)	0.09 (− 0.11, 0.29)	0.05 (− 0.15, 0.25)
*P* for trend	–	0.688	0.456	0.744
Women				
Quintile 1 (lowest adherence)	65.52 (10.05)	0.00	0.00	0.00
Quintile 2	64.24 (9.76)	− 1.28 (− 1.73, − 0.84)***	− 0.35 (− 0.75, 0.05)	− 0.35 (− 0.75, 0.05)
Quintile 3	63.88 (9.73)	− 1.64 (− 2.09, − 1.20)***	− 0.22 (− 0.62, 0.19)	− 0.23 (− 0.64, 0.17)
Quintile 4	63.27 (9.67)	− 2.25 (− 2.69, − 1.81)***	− 0.38 (− 0.78, 0.03)	− 0.40 (− 0.80, 0.01)
Quintile 5 (highest adherence)	62.62 (9.55)	− 2.90 (− 3.33, − 2.46)***	− 0.42 (− 0.83, − 0.01)^*^	− 0.46 (− 0.88, − 0.05)^*^
Per 10-point increment	63.87 (9.79)	− 0.85 (− 0.97, − 0.73)***	− 0.11 (− 0.23, 0.0003)	− 0.13 (− 0.24, − 0.01)^*^
*P* for trend	–	< 0.001	0.070	0.041

SD, standard deviation, CI, confidence interval

P for interaction between sex and planetary health diet scores on heart age was lower than 0.001

Model 1: adjusted for sex (for all participants only), education, occupation, family income, alcohol use, physical activity, self-rated health, objective health status and recruitment phase

Model 2: additionally adjusted for body mass index

^*^P < 0.05, **P < 0.01, ^***^P < 0.001

### Sensitivity analyses

Sensitivity analysis after excluding deaths occurring within the first two years of follow-up showed similar results (Supplementary Table 5). No significant interactions were found between PHD scores and sex, age or BMI regarding mortality risks (P_interaction_ from 0.142 to 0.967, Supplementary Table 6). Moreover, analyses using data from the first two phases, which included more precise dietary information, showed similar associations of PHD scores with mortality risks and heart age compared to analyses using data from all the three phases (Supplementary Tables 7, 8 and Supplementary Fig. 3).

## Discussion

In this large population-based prospective cohort study of middle-aged to older Chinese, we found that higher adherence to PHD was linearly associated with lower risks of all-cause and CVD mortality, but not cancer mortality, with a 10-point increase in PHD scores associating with a 6% reduction in all-cause mortality and an 8% reduction in CVD mortality. Additionally, our mediation analysis identified WBC, waist-to-hip ratio and waist-to-hip-to-height ratio as significant mediators in the relationships between PHD scores and all-cause or CVD mortality, suggesting that inflammatory and central obesity indicators may play crucial roles in these associations. Moreover, we observed a sex-specific effect on heart age, with higher PHD scores associating with lower heart age in women but not in men, indicating the potential differential impacts of dietary patterns on cardiovascular health by sex. These findings highlight the potential benefits of PHD in improving longevity and reducing predicted CVD risk, particularly in women.

Previous studies consistently showed a linear association between higher adherence to PHD and lower CVD mortality [8, 9, 13, 15]. Among these studies, only one study was conducted in Asia [13]. Since this study included Singaporean Chinese, whose dietary habits may have already changed from traditional Chinese diet, the PHD developed in this study might not be directly applicable to mainland Chinese residents. Our mediation analysis indicated that the association between PHD scores and CVD mortality was partially mediated by WBC and waist-to-hip-to-height ratio, indicating that PHD may reduce CVD mortality through its anti-inflammatory effects and reduction of central obesity. Abdominal fat accumulation was also considered as a form of systemic chronic inflammation [40]. Chronic inflammation was both risk factor and pathogenic mechanism in CVD, with the release of pro-inflammatory cytokines and the chemotaxis of inflammatory cells being primary drivers of atherosclerosis [41]. Plant-based diets was associated with lower inflammatory levels, including obesity-related inflammatory profiles [42], likely due to the presence of phytochemicals and dietary fiber [43, 44]. Furthermore, as a comprehensive plant-based diet, the PHD combines various phytochemicals that may exert synergistic anti-inflammatory effects by increasing antioxidant capacity, interacting with the gut microbiome or targeting common signaling pathways [45]. These effects could lead to lower inflammatory and abdominal fat levels, thereby reducing CVD mortality.

Previous studies suggested that PHD may have potential cardioprotective effect by improving lipid profiles, lowering blood pressure, or mitigating obesity levels [18, 21, 46]. In our cross-sectional study, we found that higher adherence to PHD was associated with lower triglycerides, WBC, waist-to-hip ratio and waist-to-hip-to-height ratio, but higher BMI, as well as mixed associations with blood pressure. The discrepancy in BMI findings may be attributed to reverse causality, where individuals with higher BMI may alter their dietary habits such as reducing meat consumption or increasing plant-based foods intake, thereby aligning more closely with the PHD. Additionally, the mix-direction association observed for SBP and DBP suggest that the mechanisms underlying the cardioprotective effects of PHD could be more complex. Notably, we found a negative association between higher PHD scores and lower WBC. Given the lack of previous studies exploring the association between PHD and WBC, our findings indicated a potential anti-inflammatory impact of PHD, warranting further investigation for clarification.

Although PHD shows potential beneficial effects on certain CVD risk factors, limited studies have explored the association between PHD and overall predicted cardiovascular health. To date, we found only one cross-sectional study of 14,155 Brazilians assessing the association between PHD scores and “ideal cardiovascular health (ICH)”, a composite score that included six risk factors (blood pressure, fasting plasma glucose, total cholesterol, BMI, smoking and physical activity) to predict cardiovascular health [18]. In this study, those in the 5th (highest) PHD quintile had an ICH 8.05% (relative predicted score differences (rPSD) (95% CI) 8.05% (5.54–10.62%)) higher than those in the first (lowest) PHD quintile, indicating the potential cardioprotective impact of PHD [18]. To enhance communication regarding CVD risk, we calculated heart age based on a 10-year CVD risk prediction model and examined the association between PHD scores and heart age. Our findings indicated a sex-specific association, with a negative association observed only in women. One possible explanation is that, compared to men, women had lower skeletal muscle mass and higher body fat content [47]. Since higher adherence to PHD is related to lower saturated fats and sugar intake, it may have a more pronounced impact on the health outcomes of women. This was supported by one previous study showing that women may derive greater health benefits from similar levels of healthy dietary intake than men [48]. Another explanation is that we included participants aged 50 years or older, most of whom were post-menopause. Notably, menopausal women experience an increased risk of CVD [49]. As estrogen might have an anti-atherogenic effect and protect the arteries from high pressure-induced damage [50, 51], the dramatic decline in estrogen levels during menopause may render the cardiovascular system more vulnerable in women aged 50 + years. Additionally, it has been reported that after the age of 50, sex-specific differences in CVD risk factors diminish or even trend unfavorably for women [52]. Given the increased cardiovascular vulnerability in this demographic, the plant-based PHD is more likely to have significant cardioprotective impact on women.

Furthermore, consistent with most previous studies [8, 13, 15, 17], our study showed the linear association between higher adherence to PHD and lower risk of all-cause mortality. The association of PHD scores with all-cause mortality and corresponding mediation analyses results were similar to those observed with CVD mortality, suggesting that the reduction in all-cause mortality was mainly due to decreased CVD mortality. No association of PHD and all cancer mortality was found in our study, expect for the 2nd and 4th PHD quintiles. Previous studies on the association between PHD and all cancer morality showed inconsistent results, with one study in Sweden showing a linear negative association [8], while others showing no association or significant association only in certain PHD groups [13, 15], possibly due to different PHD score grouping methods and variations in specific food types. Another explanation may lie in the differences in the cancer spectrum across different regions. According to the latest report from International Agency for Research on Cancer (IARC), lung cancer ranks first in cancer incidence in China, while in Sweden, prostate, breast and colorectal cancer are among the top three [53]. For example, colorectal cancer risk was highly related to dietary factors, such as the intake of vegetables, fruits, red meat and processed meat [54]. Further studies with large sample sizes on certain cancer deaths are needed to elucidate the impact of PHD on specific types of cancer mortality.

The strengths of our study included the population-based design, large sample size, long follow-up duration, comprehensive dietary assessment, and reliable death ascertainment through the Death Registry. Additionally, the use of mediation analysis to identify potential mediators and the calculation of heart age to convert relative risk into absolute risk for better risk communication are significant strengths. However, our study also has several limitations. First, the simplification of the FFQ used in phase 3 limited the accurate calculation of food consumption, such as daily energy intake, and thus we used estimation methods to harmonize dietary information from all three phases. However, sensitivity analyses using precise dietary information from the first two phases showed consistent results. Second, measurement error is inevitable in nutritional epidemiology studies, despite the FFQ used in the present study has been validated in Chinese population [28]. Third, only a single-time dietary assessment was conducted in our study, which may not capture dietary changes during the long-term follow-up. However, our previous GBCS findings showed relatively stable dietary pattern in older Chinese [55]. Fourth, although baseline data collection included dietary information and factors related to metabolic health, inflammation and obesity, the FFQ assessed the food intake over the past week and might reflect a long-term dietary behavior. Fifth, reverse causality may influence risk estimates, as participants with poor health may alter their dietary habits. However, sensitivity analyses excluding deaths within the first two years of follow-up showed consistent results, suggesting that reverse causality, if any, may not be a major concern. Finally, despite adjusting for multiple potential confounders, residual confounding from unmeasured factors could not be entirely ruled out.

## Conclusions

In conclusion, higher adherence to PHD was linearly associated with lower all-cause and CVD mortality, but not cancer mortality in Chinese aged 50 years or older, and with lower heart age in women only. Our findings support the adoption of PHD in middle-aged to older Chinese, particularly women to improve cardiovascular health.

## Supplementary Information

Below is the link to the electronic supplementary material.

Supplementary Material 1

Supplementary Material 2

## Data Availability

Because of ethical restrictions protecting patient privacy, data are available on request only from the Guangzhou Biobank Cohort Study Data Access Committee. Please contact us at gbcsdata@hku.hk for fielding data accession requests.
