# Supplementary material for: Planetary health diet: dissecting the link between diet, mortality risk and heart age from a 16-year follow-up of the Guangzhou Biobank Cohort Study
Source: Eur J Nutr. 2026 May 26;65(4):140. doi: 10.1007/s00394-026-04002-x (PMC13212740; doi:10.1007/s00394-026-04002-x)
Supplement: Supplementary file 1 — Supplementary Material 1 [file 394_2026_4002_MOESM1_ESM.docx]

**Planetary health diet: dissecting the link between diet, mortality risk and heart age from a 16-year follow-up of the Guangzhou Biobank Cohort Study**

***Journal:*** **European Journal of Nutrition**

Ting Yu Lu^1,5^, Jiao Wang, PhD^1,5^, Ying Yue Huang^1,5^, Wen Bo Tian^1,5^, Ya Li Jin, MPhil^2^, Tai Hing Lam, MD^2,3,5^, Wei Sen Zhang, PhD^2,5*^, Lin Xu, PhD^1,3,4,5*^

^1^ School of Public Health, Sun Yat-sen University, Guangzhou 510080, China

^2^ Guangzhou Twelfth People’s Hospital, Guangzhou 510620, China

^3^ School of Public Health, the University of Hong Kong, Hong Kong

^4^ Institute of Applied Health Research, University of Birmingham, Birmingham B15 2TT, UK

^5^ Greater Bay Area Public Health Research Collaboration, China

^*^ Joint corresponding authors

Corresponding author (1): Professor L Xu

School of Public Health, Sun Yat-sen University,

74 Zhongshan 2nd Road, Guangzhou, Guangdong Province, China

Tel: (86) 20-87335523

Fax: (86) 20-87330446

Email: xulin27@mail.sysu.edu.cn

Corresponding author (2): Professor WS Zhang

Guangzhou Twelfth People’s Hospital, Guangzhou 510620, China

Tel: (86) 20-38665762

Email: zwsgzcn@163.com

**Contents**

**Supplementary Table 1** Scoring criteria for each food group in planetary health diet (PHD) based on daily energy intake of 2500 kcal/day

**Supplementary Table 2** Representative food items within each food category of the planetary health diet in Guangzhou Biobank Cohort Study

**Supplementary Table 3** Associations of planetary health diet scores with metabolic health, inflammation and obesity indicators in Guangzhou Biobank Cohort Study (GBCS) participants in 2003-2008

**Supplementary Table 4** Mediation proportions of metabolic health, inflammation and obesity indicators for the associations of baseline planetary health diet scores with all-cause or cardiovascular disease mortality in Guangzhou Biobank Cohort Study (GBCS) participants in 2003-2008 and followed up till November 2023

**Supplementary Table 5** Associations of baseline planetary health diet scores with all-cause, cardiovascular disease and cancer mortality on 25,366 Guangzhou Biobank Cohort Study (GBCS) participants excluding deaths occurring within the first two years of follow-up in 2003-2008 and followed up till November 2023

**Supplementary Table 6** Associations of baseline planetary health diet scores with all-cause, cardiovascular disease, cancer mortality and heart age stratified by selected baseline characteristics in Guangzhou Biobank Cohort Study (GBCS) participants

**Supplementary Table 7** Associations of baseline planetary health diet scores with all-cause, cardiovascular disease and cancer mortality on 17,690 Guangzhou Biobank Cohort Study (GBCS) participants in 2003-2006 and followed up till November 2023

**Supplementary Table 8** Associations of planetary health diet scores with heart age on 18,257 Guangzhou Biobank Cohort Study (GBCS) participants without baseline cardiovascular disease in 2003-2006 and stratified by sex

**Supplementary Figure 1** Flow chart of the study sample selection in the main analysis in Guangzhou Biobank Cohort Study (GBCS)

**Supplementary Figure 2** Timeline and the number of participants in the main analysis in Guangzhou Biobank Cohort Study

**Supplementary Figure 3** Restricted cubic spline plots for the associations of baseline planetary health diet scores with all-cause, cardiovascular disease and cancer mortality on 17,690 Guangzhou Biobank Cohort Study (GBCS) participants in 2003-2006 and followed up till November 2023

**Supplementary Table 1 Scoring criteria for each food group in planetary health diet (PHD) based on daily energy intake of 2500 kcal/day**

|  | **Recommended intake (g/day)** | **Planetary diet health score, point** | | | |
| --- | --- | --- | --- | --- | --- |
|  |  | **Score=0** | **0<score<10** | **Score=10** | **10>score>0** |
| **Emphasized food** | | | | | |
| Whole grains | 232 | Intake=0 | 0<intake<232 | Intake≥232 | - |
| All vegetables | 300 (200-600) | Intake=0 | 0<intake<300 | Intake≥300 | - |
| Fruits | 200 (100-300) | Intake=0 | 0<intake<200 | Intake≥200 | - |
| Legumes | 75 (0-100) | Intake=0 | 0<intake<75 | Intake≥75 | - |
| Nuts | 50 (0-75) | Intake=0 | 0<intake<50 | Intake≥50 | - |
| **Optional food** |  |  |  |  |  |
| Poultry | 29 (0-58) | Intake=0, or intake≥58 | 0<intake<29 | Intake=29 | 29<intake<58 |
| Dairy foods | 250 (0-500) | Intake=0, or intake≥500 | 0<intake<250 | Intake=250 | 250<intake<500 |
| Eggs | 13 (0-25) | Intake=0, or intake≥25 | 0<intake<13 | Intake=13 | 13<intake<25 |
| Fish | 28 (0-100) | Intake=0, or intake≥100 | 0<intake<28 | Intake=28 | 28<intake<100 |
| Unsaturated oil | 40 (20-80) | Intake=0, or intake≥80 | 0<intake<40 | Intake=40 | 40<intake<80 |
| Tuber and starchy vegetables | 50 (0-100) | Intake=0, or intake≥100 | 0<intake<50 | Intake=50 | 50<intake<100 |
| **Limited food** |  |  |  |  |  |
| Red meat | 14 (0-28) | Intake≥28 | - | 0≤intake≤14 | 14<intake<28 |
| Saturated oil | 11.8 (0-11.8) | Intake≥11.8 | - | Intake=0 | 0<intake<11.8 |
| Added sugar | 31 (0-31) | Intake≥31 | - | Intake=0 | 0<intake<31 |

**Supplementary Table 2 Representative food items within each food category of the planetary health diet in Guangzhou Biobank Cohort Study**

| **Food Category in the PHD** | **Representative food items in the GBCS** |
| --- | --- |
| Whole grains | Oat, corn, whole wheat bread |
| All vegetables | Chinese leaf, watercress, spinach |
| Fruits | Orange, apple, banana |
| Legumes | Red bean, brow bean, lentils |
| Nuts | Peanut, walnut, almond |
| Poultry | Turkey, chicken, duck |
| Dairy foods | Whole milk, skim milk, milk powder |
| Eggs | Boiled egg, fried egg, scrambled egg |
| Fish | Ribbon fish, grass fish, Sardines |
| Unsaturated oil | Corn oil, peanut oil, olive oil |
| Tuber and starchy vegetables | Sweet potato, potato, lotus root |
| Red meat | Pork, lamb, beef |
| Saturated oil | Lard, butter |
| Added sugar | White sugar, syrup |

PHD=planetary health diet, GBCS=Guangzhou Biobank Cohort Study.

**Supplementary Table 3 Associations of planetary health diet scores with metabolic health, inflammation and obesity indicators in Guangzhou Biobank Cohort Study (GBCS) participants in 2003-2008**

|  | **Planetary health diet score (range: 0-140), β (95% CI)** | | | | | **P for trend** |
| --- | --- | --- | --- | --- | --- | --- |
|  | **Quintile 1**  **(lowest adherence)** | **Quintile 2** | **Quintile 3** | **Quintile 4** | **Quintile 5**  **(highest adherence)** |  |
| Fasting glucose, mmol/L | | | |  |  |  |
| Crude model | 0.00 | 0.02 (-0.02, 0.07) | 0.01 (-0.04, 0.05) | 0.01 (-0.04, 0.05) | 0.01 (-0.03, 0.06) | 0.802 |
| Model 1 | 0.00 | 0.02 (-0.03, 0.06) | 0.01 (-0.03, 0.06) | 0.01 (-0.04, 0.05) | 0.01 (-0.04, 0.05) | 0.933 |
| LDL-C, mmol/L | | | |  |  |  |
| Crude model | 0.00 | 0.02 (-0.004, 0.05) | 0.04 (0.01, 0.07) ^**^ | 0.07 (0.05, 0.10) ^***^ | 0.08 (0.05, 0.10) ^***^ | <0.001 |
| Model 1 | 0.00 | -0.004 (-0.03, 0.02) | 0.005 (-0.02, 0.03) | 0.02 (-0.005, 0.05) | -0.01 (-0.03, 0.02) | 0.620 |
| HDL-C, mmol/L | | | | | | |
| Crude model | 0.00 | -0.003 (-0.02, 0.01) | -0.004 (-0.02, 0.01) | 0.01 (-0.004, 0.03) | 0.01 (-0.003, 0.03) | 0.027 |
| Model 1 | 0.00 | -0.0005 (-0.02, 0.01) | -0.005 (-0.02, 0.01) | 0.01 (-0.01, 0.02) | 0.005 (-0.01, 0.02) | 0.337 |
| Triglycerides, mmol/L | | | | | | |
| Crude model | 0.00 | 0.01 (-0.03, 0.04) | -0.001 (-0.04, 0.03) | 0.02 (-0.02, 0.05) | -0.02 (-0.05, 0.02) | 0.520 |
| Model 1 | 0.00 | -0.01 (-0.05, 0.02) | -0.02 (-0.06, 0.01) | -0.01 (-0.04, 0.03) | -0.07 (-0.11, -0.03) ^***^ | 0.002 |
| Total cholesterol, mmol/L | | | | | | |
| Crude model | 0.00 | -0.01 (-0.05, 0.03) | -0.01 (-0.05, 0.03) | 0.05 (0.01, 0.09) ^*^ | 0.03 (-0.01, 0.07) | 0.008 |
| Model 1 | 0.00 | -0.01 (-0.05, 0.03) | -0.02 (-0.06, 0.03) | 0.02 (-0.02, 0.07) | -0.01 (-0.06, 0.03) | 0.872 |
| Systolic blood pressure, mmHg | | | | | | |
| Crude model | 0.00 | 0.15 (-0.65, 0.96) | -0.60 (-1.40, 0.21) | -2.06 (-2.86, -1.25) ^***^ | -2.36 (-3.16, -1.55) ^***^ | <0.001 |
| Model 1 | 0.00 | 0.83 (0.05, 1.61) ^*^ | 0.40 (-0.39, 1.18) | -0.58 (-1.37, 0.22) | -0.42 (-1.24, 0.40) | 0.016 |
| Diastolic blood pressure, mmHg | | | | | | |
| Crude model | 0.00 | 0.29 (-0.12, 0.70) | -0.07 (-0.48, 0.34) | -0.69 (-1.10, -0.28) ^**^ | -0.74 (-1.14, -0.33) ^***^ | <0.001 |
| Model 1 | 0.00 | 0.35 (-0.06, 0.75) | 0.01 (-0.40, 0.42) | -0.43 (-0.85, -0.02) ^*^ | -0.42 (-0.85, 0.002) | 0.001 |
| White blood cell count, 10^9^/L | | | | | | |
| Crude model | 0.00 | -0.04 (-0.10, 0.02) | -0.10 (-0.15, -0.04) ^**^ | -0.15 (-0.21, -0.10) ^***^ | -0.15 (-0.21, -0.10) ^***^ | <0.001 |
| Model 1 | 0.00 | -0.02 (-0.08, 0.04) | -0.05 (-0.10, 0.01) | -0.09 (-0.14, -0.03) ^**^ | -0.06 (-0.12, -0.01) ^*^ | 0.004 |
| hsCRP, mg/L | | | | | | |
| Crude model | 0.00 | -0.19 (-0.35, -0.03) ^*^ | -0.14 (-0.31, 0.02) | -0.29 (-0.45, -0.13) ^***^ | -0.25 (-0.41, -0.09) ^**^ | 0.001 |
| Model 1 | 0.00 | -0.10 (-0.26, 0.06) | 0.01 (-0.16, 0.17) | -0.12 (-0.28, 0.04) | -0.05 (-0.21, 0.12) | 0.525 |
| Albumin, g/L | | | | | | |
| Crude model | 0.00 | 0.19 (0.02, 0.36) ^*^ | 0.17 (-0.01, 0.34) | 0.24 (0.06, 0.41) ^**^ | 0.36 (0.18, 0.54) ^***^ | <0.001 |
| Model 1 | 0.00 | 0.09 (-0.08, 0.26) | 0.04 (-0.14, 0.21) | 0.11 (-0.07, 0.28) | 0.20 (0.01, 0.38) ^*^ | 0.057 |
| Body mass index, kg/m^2^ | |  |  |  |  |  |
| Crude model | 0.00 | 0.15 (0.03, 0.27) ^*^ | 0.18 (0.06, 0.30) ^**^ | 0.22 (0.10, 0.34) ^***^ | 0.39 (0.27, 0.51) ^***^ | <0.001 |
| Model 1 | 0.00 | 0.11 (-0.01, 0.24) | 0.17 (0.04, 0.29) ^**^ | 0.22 (0.10, 0.35) ^**^ | 0.35 (0.22, 0.48) ^***^ | <0.001 |
| Waist circumference, cm | |  |  |  |  |  |
| Crude model | 0.00 | -0.10 (-0.43, 0.23) | -0.35 (-0.68, -0.02) ^*^ | -0.62 (-0.95, -0.29) ^***^ | -0.67 (-1.00, -0.34) ^***^ | <0.001 |
| Model 1 | 0.00 | 0.09 (-0.24, 0.41) | 0.03 (-0.29, 0.36) | 0.04 (-0.29, 0.37) | 0.15 (-0.19, 0.49) | 0.527 |
| Waist-to-hip ratio |  |  |  |  |  |  |
| Crude model | 0.00 | -0.002 (-0.005, 0.0001) | -0.005 (-0.008, -0.003) ^***^ | -0.009 (-0.012, -0.007) ^***^ | -0.011 (-0.013, -0.009) ^***^ | <0.001 |
| Model 1 | 0.00 | -0.0003 (-0.003, 0.002) | -0.002 (-0.004, 0.001) | -0.003 (-0.006, -0.001) ^*^ | -0.003 (-0.005, -0.001) ^*^ | 0.002 |
| Waist-to-height ratio | |  |  |  |  |  |
| Crude model | 0.00 | -0.001 (-0.003, 0.001) | -0.003 (-0.005, -0.001) ^*^ | -0.004 (-0.006, -0.002) ^***^ | -0.004 (-0.006, -0.002) ^***^ | <0.001 |
| Model 1 | 0.00 | 0.0001 (-0.002, 0.002) | -0.0002 (-0.002, 0.002) | -0.0004 (-0.003, 0.002) | -0.0001 (-0.002, 0.002) | 0.770 |
| Waist-to-hip-to-height ratio, m^-1^ | |  |  |  |  |  |
| Crude model | 0.00 | -0.002 (-0.004, -0.0002) ^*^ | -0.004 (-0.006, -0.002) ^***^ | -0.006 (-0.008, -0.004) ^***^ | -0.007 (-0.009, -0.005) ^***^ | <0.001 |
| Model 1 | 0.00 | -0.001 (-0.002, 0.001) | -0.002 (-0.003, 0.00005) | -0.003 (-0.005, -0.001) ^**^ | -0.003 (-0.004, -0.001) ^***^ | <0.001 |
| Body roundness index | |  |  |  |  |  |
| Crude model | 0.00 | -0.02 (-0.06, 0.02) | -0.06 (-0.10, -0.02) ^**^ | -0.08 (-0.12, -0.04) ^***^ | -0.08 (-0.12, -0.04) ^***^ | <0.001 |
| Model 1 | 0.00 | -0.001 (-0.04, 0.04) | -0.01 (-0.05, 0.03) | -0.01 (-0.05, 0.03) | -0.004 (-0.05, 0.04) | 0.709 |

LDL-C=low-density lipoprotein cholesterol, HDL-C=high-density lipoprotein cholesterol, hsCRP=hypersensitive C-reactive protein, CI=confidence interval.

Model 1: adjusted for sex, age, education, occupation, family income, smoking status, alcohol use, physical activity, self-rated health, objective health status, recruitment phase and body mass index (except for obesity indicators).

^*^P<0.05, ^**^P<0.01, ^***^P<0.001.

**Supplementary Table 4 Mediation proportions of metabolic health, inflammation and obesity indicators for the associations of baseline planetary health diet scores with all-cause or cardiovascular disease mortality in Guangzhou Biobank Cohort Study (GBCS) participants in 2003-2008 and followed up till November 2023**

|  | **HR (95% CI) ^a^** | | **Mediation proportion (95% CI) (%)** | **P value** |
| --- | --- | --- | --- | --- |
|  | **Model without potential mediator** | **Model with potential mediator** |  |  |
| **All-cause mortality** | | | | |
| Triglycerides ^b^ |  |  |  |  |
| Per 10-point increment | 0.91 (0.89, 0.93) | 0.91 (0.89, 0.93) | - | - |
| White blood cell count |  |  |  |  |
| Per 10-point increment | 0.91 (0.89, 0.93) | 0.92 (0.90, 0.94) | 6.2 (3.2, 11.7) | <0.001 |
| Body mass index ^b^ |  |  |  |  |
| Per 10-point increment | 0.91 (0.89, 0.93) | 0.91 (0.89, 0.93) | - | - |
| Waist-to-hip ratio |  |  |  |  |
| Per 10-point increment | 0.91 (0.89, 0.93) | 0.91 (0.89, 0.93) | 2.6 (0.9, 7.2) | 0.026 |
| Waist-to-hip-to-height ratio | |  |  |  |
| Per 10-point increment | 0.91 (0.89, 0.93) | 0.92 (0.90, 0.94) | 5.4 (2.8, 9.9) | <0.001 |
| **Cardiovascular disease mortality** | | | | |
| Triglycerides ^b^ |  |  |  |  |
| Per 10-point increment | 0.89 (0.86, 0.92) | 0.89 (0.86, 0.92) | - | - |
| White blood cell count |  |  |  |  |
| Per 10-point increment | 0.89 (0.86, 0.92) | 0.90 (0.86, 0.93) | 7.9 (4.1, 14.9) | <0.001 |
| Body mass index ^b^ |  |  |  |  |
| Per 10-point increment | 0.89 (0.86, 0.92) | 0.88 (0.85, 0.92) | - | - |
| Waist-to-hip ratio ^c^ |  |  |  |  |
| Per 10-point increment | 0.89 (0.86, 0.92) | 0.89 (0.86, 0.92) | 2.9 (0.5, 16.2) | 0.130 |
| Waist-to-hip-to-height ratio | |  |  |  |
| Per 10-point increment | 0.89 (0.86, 0.92) | 0.90 (0.86, 0.93) | 7.4 (3.0, 17.0) | 0.006 |

HR=hazard ratio, CI=confidence interval.

^a^ HRs (95% CIs) were adjusted for sex and age.

^b^ The proportion of mediation effect was too small to calculate reliably, indicating this factor was not intermediate to planetary health diet scores.

^c^ Since the transform that the SAS “mediate” macro used to get the CI for mediation proportion did not go below zero, the CI did not contain the null value (0) even the P value for mediation analysis was not significant. According to the guidance of the “mediate” macro in SAS, we need to pay attention to the P value rather than the CI.

**Supplementary Table 5 Associations of baseline planetary health diet scores with all-cause, cardiovascular disease and cancer mortality on 25,366 Guangzhou Biobank Cohort Study (GBCS) participants excluding deaths occurring within the first two years of follow-up in 2003-2008 and followed up till November 2023**

|  | **Planetary health diet score (range: 0-140), HR (95% CI)** | | | | | | **P for trend** |
| --- | --- | --- | --- | --- | --- | --- | --- |
|  | **Quintile 1**  **(lowest adherence)** | **Quintile 2** | **Quintile 3** | **Quintile 4** | **Quintile 5**  **(highest adherence)** | **Per 10-point increment** |  |
| Person-years | 83,277 | 83,508 | 83,154 | 83,546 | 83,893 | 417,376 | - |
| All-cause mortality |  |  |  |  |  |  |  |
| No. of deaths | 1621 | 1340 | 1292 | 1145 | 1025 | 6423 | - |
| Mortality rate,  per 10,000 person-years | 194.7 | 160.5 | 155.4 | 137.1 | 122.2 | 153.9 | - |
| Crude model | 1.00 | 0.83 (0.77, 0.89) ^***^ | 0.80 (0.75, 0.86) ^***^ | 0.71 (0.66, 0.76) ^***^ | 0.64 (0.59, 0.69) ^***^ | 0.87 (0.86, 0.89) ^***^ | <0.001 |
| Model 1 | 1.00 | 0.89 (0.83, 0.96) ^**^ | 0.92 (0.85, 0.99) ^*^ | 0.85 (0.79, 0.92) ^***^ | 0.83 (0.76, 0.90) ^***^ | 0.95 (0.92, 0.97) ^***^ | <0.001 |
| Model 2 | 1.00 | 0.89 (0.83, 0.96) ^**^ | 0.92 (0.85, 0.99) ^*^ | 0.85 (0.78, 0.92) ^***^ | 0.82 (0.76, 0.90) ^***^ | 0.95 (0.92, 0.97) ^***^ | <0.001 |
| Cardiovascular disease mortality | |  |  |  |  |  |  |
| No. of deaths | 654 | 553 | 499 | 439 | 377 | 2522 | - |
| Mortality rate,  per 10,000 person-years | 78.5 | 66.2 | 60.0 | 52.5 | 44.9 | 60.4 | - |
| Crude model | 1.00 | 0.85 (0.76, 0.95) ^**^ | 0.77 (0.68, 0.86) ^***^ | 0.68 (0.60, 0.76) ^***^ | 0.59 (0.52, 0.66) ^***^ | 0.85 (0.82, 0.88) ^***^ | <0.001 |
| Model 1 | 1.00 | 0.95 (0.84, 1.07) | 0.89 (0.78, 1.002) | 0.82 (0.72, 0.93) ^**^ | 0.78 (0.68, 0.89) ^***^ | 0.92 (0.89, 0.96) ^***^ | <0.001 |
| Model 2 | 1.00 | 0.95 (0.84, 1.06) | 0.88 (0.78, 0.99) ^*^ | 0.81 (0.71, 0.92) ^**^ | 0.77 (0.67, 0.88) ^***^ | 0.92 (0.89, 0.95) ^***^ | <0.001 |
| Cancer mortality |  |  |  |  |  |  |  |
| No. of deaths | 474 | 366 | 420 | 351 | 335 | 1946 | - |
| Mortality rate,  per 10,000 person-years | 56.9 | 43.8 | 50.5 | 42.0 | 39.9 | 46.6 | - |
| Crude model | 1.00 | 0.77 (0.67, 0.88) ^***^ | 0.89 (0.78, 1.01) | 0.74 (0.64, 0.84) ^***^ | 0.70 (0.61, 0.80) ^***^ | 0.90 (0.87, 0.94) ^***^ | <0.001 |
| Model 1 | 1.00 | 0.80 (0.70, 0.93) ^**^ | 0.98 (0.85, 1.12) | 0.85 (0.74, 0.99) ^*^ | 0.88 (0.75, 1.02) | 0.97 (0.93, 1.01) | 0.177 |
| Model 2 | 1.00 | 0.80 (0.69, 0.92) ^**^ | 0.98 (0.85, 1.12) | 0.85 (0.73, 0.98) ^*^ | 0.87 (0.75, 1.02) | 0.97 (0.93, 1.01) | 0.177 |

HR=hazard ratio, CI=confidence interval.

Model 1: adjusted for sex, age, education, occupation, family income, smoking status, alcohol use, physical activity, self-rated health, objective health status and recruitment phase.

Model 2: additionally adjusted for body mass index.

^*^P<0.05, ^**^P<0.01, ^***^P<0.001.

**Supplementary Table 6 Associations of baseline planetary health diet scores with all-cause, cardiovascular disease, cancer mortality and heart age stratified by selected baseline characteristics in Guangzhou Biobank Cohort Study (GBCS) participants**

|  | **Planetary health diet score (range: 0-140)** | | | | | **P for interaction** |
| --- | --- | --- | --- | --- | --- | --- |
|  | **Quintile 1**  **(lowest adherence)** | **Quintile 2** | **Quintile 3** | **Quintile 4** | **Quintile 5**  **(highest adherence)** |  |
| **All-cause mortality, HR (95% CI) ^a^** | | | | | | |
| Sex |  |  |  |  |  | 0.142 |
| Men | 1.00 | 0.87 (0.77, 0.97) ^*^ | 0.92 (0.82, 1.03) | 0.78 (0.69, 0.88) ^***^ | 0.73 (0.64, 0.83) ^***^ |  |
| Women | 1.00 | 0.92 (0.83, 1.02) | 0.94 (0.85, 1.04) | 0.91 (0.82, 1.01) | 0.90 (0.80, 0.99) ^*^ |  |
| Age, years |  |  |  |  |  | 0.664 |
| <65 | 1.00 | 0.92 (0.81, 1.05) | 0.89 (0.78, 1.01) | 0.81 (0.71, 0.93) ^**^ | 0.76 (0.66, 0.88) ^***^ |  |
| ≥65 | 1.00 | 0.88 (0.81, 0.97) ^**^ | 0.96 (0.88, 1.06) | 0.90 (0.82, 0.99) ^*^ | 0.86 (0.78, 0.96) ^**^ |  |
| Body mass index, kg/m^2^ | |  |  |  |  | 0.867 |
| <25 | 1.00 | 0.85 (0.78, 0.93) ^***^ | 0.91 (0.83, 1.003) | 0.84 (0.76, 0.92) ^***^ | 0.80 (0.72, 0.88) ^***^ |  |
| ≥25 | 1.00 | 0.99 (0.87, 1.14) | 0.95 (0.83, 1.09) | 0.88 (0.77, 1.02) | 0.87 (0.75, 1.005) |  |
| **Cardiovascular disease mortality, HR (95% CI) ^a^** | | | | | | |
| Sex |  |  |  |  |  | 0.773 |
| Men | 1.00 | 0.98 (0.81, 1.17) | 0.90 (0.74, 1.09) | 0.75 (0.61, 0.92) ^**^ | 0.75 (0.61, 0.94) ^*^ |  |
| Women | 1.00 | 0.95 (0.81, 1.11) | 0.89 (0.76, 1.05) | 0.86 (0.73, 1.01) | 0.80 (0.67, 0.95) ^*^ |  |
| Age, years |  |  |  |  |  | 0.195 |
| <65 | 1.00 | 1.01 (0.81, 1.26) | 0.98 (0.78, 1.22) | 0.84 (0.66, 1.06) | 0.68 (0.52, 0.88) ^**^ |  |
| ≥65 | 1.00 | 0.94 (0.82, 1.08) | 0.88 (0.76, 1.02) | 0.85 (0.73, 0.99) ^*^ | 0.83 (0.71, 0.97) ^*^ |  |
| Body mass index, kg/m^2^ | |  |  |  |  | 0.817 |
| <25 | 1.00 | 0.95 (0.82, 1.09) | 0.87 (0.75, 1.01) | 0.85 (0.73, 0.99) ^*^ | 0.80 (0.67, 0.94) ^**^ |  |
| ≥25 | 1.00 | 0.97 (0.79, 1.18) | 0.92 (0.75, 1.12) | 0.77 (0.62, 0.95) ^*^ | 0.71 (0.57, 0.90) ^**^ |  |
| **Cancer mortality, HR (95% CI) ^a^** | | | | | | |
| Sex |  |  |  |  |  | 0.185 |
| Men | 1.00 | 0.78 (0.64, 0.96) ^*^ | 1.01 (0.83, 1.23) | 0.75 (0.60, 0.94) ^*^ | 0.80 (0.64, 1.002) |  |
| Women | 1.00 | 0.86 (0.70, 1.04) | 0.98 (0.81, 1.18) | 0.95 (0.78, 1.16) | 0.96 (0.79, 1.17) |  |
| Age, years |  |  |  |  |  | 0.967 |
| <65 | 1.00 | 0.78 (0.63, 0.96) ^*^ | 0.84 (0.68, 1.03) | 0.78 (0.63, 0.97) ^*^ | 0.79 (0.63, 0.98) ^*^ |  |
| ≥65 | 1.00 | 0.85 (0.70, 1.03) | 1.13 (0.94, 1.36) | 0.93 (0.77, 1.14) | 0.97 (0.80, 1.19) |  |
| Body mass index, kg/m^2^ | |  |  |  |  | 0.904 |
| <25 | 1.00 | 0.72 (0.61, 0.86) ^***^ | 0.97 (0.83, 1.14) | 0.80 (0.68, 0.96) ^*^ | 0.85 (0.71, 1.01) |  |
| ≥25 | 1.00 | 1.09 (0.85, 1.40) | 1.04 (0.81, 1.35) | 0.99 (0.76, 1.29) | 0.99 (0.76, 1.31) |  |
| **Heart age, years, β (95% CI) ^b^** | | | | | | |
| Age, years |  |  |  |  |  | 0.191 |
| <65 | 0.00 | 0.03 (-0.26, 0.32) | -0.06 (-0.35, 0.23) | -0.26 (-0.55, 0.03) | -0.14 (-0.44, 0.16) |  |
| ≥65 | 0.00 | 0.12 (-0.33, 0.57) | -0.19 (-0.65, 0.26) | -0.33 (-0.80, 0.14) | -0.25 (-0.74, 0.24) |  |
| Body mass index, kg/m^2^ | |  |  |  |  | 0.133 |
| <25 | 0.00 | -0.07 (-0.48, 0.34) | -0.02 (-0.44, 0.39) | -0.53 (-0.95, -0.11) ^*^ | -0.27 (-0.70, 0.16) |  |
| ≥25 | 0.00 | 0.14 (-0.48, 0.77) | -0.21 (-0.84, 0.42) | 0.14 (-0.50, 0.78) | 0.11 (-0.54, 0.76) |  |

HR=hazard ratio, CI=confidence interval.

^a^ HRs (95% CIs) were adjusted for sex, age, education, occupation, family income, smoking status, alcohol use, physical activity, self-rated health, objective health status, recruitment phase and body mass index, as appropriate.

^b^ βs (95% CIs) were adjusted for sex, education, occupation, family income, alcohol use, physical activity, self-rated health, objective health status, recruitment phase and body

mass index, as appropriate.

^*^P<0.05, ^**^P<0.01, ^***^P<0.001.

**Supplementary Table 7 Associations of baseline planetary health diet scores with all-cause, cardiovascular disease and cancer mortality on 17,690 Guangzhou Biobank Cohort Study (GBCS) participants in 2003-2006 and followed up till November 2023**

|  | **Planetary health diet score (range: 0-140), HR (95% CI)** | | | | | | **P for trend** |
| --- | --- | --- | --- | --- | --- | --- | --- |
|  | **Quintile 1**  **(lowest adherence)** | **Quintile 2** | **Quintile 3** | **Quintile 4** | **Quintile 5**  **(highest adherence)** | **Per 10-point increment** |  |
| Person-years | 58,405 | 59,533 | 59,604 | 59,269 | 60,337 | 297,147 | - |
| All-cause mortality |  |  |  |  |  |  |  |
| No. of deaths | 1219 | 1080 | 1036 | 974 | 941 | 5250 | - |
| Mortality rate,  per 10,000 person-years | 208.7 | 181.4 | 173.8 | 164.3 | 156.0 | 176.7 | - |
| Crude model | 1.00 | 0.86 (0.79, 0.93) ^***^ | 0.81 (0.75, 0.88) ^***^ | 0.77 (0.70, 0.83) ^***^ | 0.72 (0.66, 0.79) ^***^ | 0.91 (0.89, 0.93) ^***^ | <0.001 |
| Model 1 | 1.00 | 0.91 (0.83, 0.99) ^*^ | 0.90 (0.83, 0.98) ^*^ | 0.87 (0.80, 0.95) ^**^ | 0.83 (0.76, 0.91) ^***^ | 0.95 (0.93, 0.97) ^***^ | <0.001 |
| Model 2 | 1.00 | 0.90 (0.83, 0.98) ^*^ | 0.90 (0.83, 0.98) ^*^ | 0.86 (0.79, 0.94) ^**^ | 0.83 (0.76, 0.91) ^***^ | 0.95 (0.93, 0.97) ^***^ | <0.001 |
| Cardiovascular disease mortality | |  |  |  |  |  |  |
| No. of deaths | 478 | 454 | 409 | 375 | 345 | 2061 | - |
| Mortality rate,  per 10,000 person-years | 81.8 | 76.3 | 68.6 | 63.3 | 57.2 | 69.4 | - |
| Crude model | 1.00 | 0.92 (0.81, 1.04) | 0.82 (0.71, 0.93) ^**^ | 0.75 (0.65, 0.86) ^***^ | 0.67 (0.58, 0.77) ^***^ | 0.88 (0.85, 0.92) ^***^ | <0.001 |
| Model 1 | 1.00 | 0.998 (0.88, 1.14) | 0.93 (0.81, 1.06) | 0.86 (0.75, 0.98) ^*^ | 0.80 (0.69, 0.92) ^**^ | 0.93 (0.89, 0.97) ^***^ | <0.001 |
| Model 2 | 1.00 | 0.997 (0.87, 1.14) | 0.92 (0.81, 1.06) | 0.86 (0.74, 0.98) ^*^ | 0.79 (0.69, 0.92) ^**^ | 0.93 (0.89, 0.96) ^***^ | <0.001 |
| Cancer mortality |  |  |  |  |  |  |  |
| No. of deaths | 352 | 301 | 296 | 285 | 308 | 1542 | - |
| Mortality rate,  per 10,000 person-years | 60.3 | 50.6 | 49.7 | 48.1 | 51.0 | 51.9 | - |
| Crude model | 1.00 | 0.84 (0.72, 0.97) ^*^ | 0.82 (0.70, 0.95) ^*^ | 0.79 (0.68, 0.92) ^**^ | 0.84 (0.72, 0.98) ^*^ | 0.95 (0.91, 0.99) ^*^ | 0.019 |
| Model 1 | 1.00 | 0.85 (0.73, 0.99) ^*^ | 0.86 (0.74, 1.01) | 0.85 (0.73, 1.003) | 0.91 (0.78, 1.07) | 0.97 (0.93, 1.02) | 0.311 |
| Model 2 | 1.00 | 0.84 (0.72, 0.99) ^*^ | 0.86 (0.74, 1.01) | 0.85 (0.73, 1.001) | 0.91 (0.77, 1.07) | 0.97 (0.93, 1.02) | 0.301 |

HR=hazard ratio, CI=confidence interval.

Model 1: adjusted for sex, age, education, occupation, family income, smoking status, alcohol use, physical activity, self-rated health and objective health status.

Model 2: additionally adjusted for body mass index.

^*^P<0.05, ^**^P<0.01, ^***^P<0.001.

**Supplementary Table 8 Associations of planetary health diet scores with heart age on 18,257 Guangzhou Biobank Cohort Study (GBCS) participants without baseline cardiovascular disease in 2003-2006 and stratified by sex**

|  | **Heart age, mean (SD), years** | **Heart age, years, β (95% CI)** | | |
| --- | --- | --- | --- | --- |
|  |  | **Crude model** | **Model 1** | **Model 2** |
| **All participants** | | | | |
| Quintile 1  (lowest adherence) | 67.33 (9.98) | 0.00 | 0.00 | 0.00 |
| Quintile 2 | 66.69 (9.98) | -0.64 (-1.09, -0.19) ^**^ | -0.26 (-0.66, 0.14) | -0.24 (-0.64, 0.16) |
| Quintile 3 | 66.35 (9.75) | -0.98 (-1.43, -0.53) ^***^ | -0.43 (-0.83, -0.02) ^*^ | -0.43 (-0.83, -0.02) ^*^ |
| Quintile 4 | 66.15 (9.78) | -1.18 (-1.63, -0.72) ^***^ | -0.34 (-0.74, 0.07) | -0.37 (-0.78, 0.03) |
| Quintile 5  (highest adherence) | 66.13 (9.69) | -1.19 (-1.64, -0.74) ^***^ | -0.19 (-0.60, 0.22) | -0.25 (-0.65, 0.16) |
| Per 10-point increment | 66.53 (9.85) | -0.33 (-0.46, -0.21) ^***^ | -0.03 (-0.14, 0.09) | -0.04 (-0.16, 0.07) |
| P for trend | - | <0.001 | 0.333 | 0.191 |
| **Men** | | | | |
| Quintile 1  (lowest adherence) | 70.06 (9.50) | 0.00 | 0.00 | 0.00 |
| Quintile 2 | 70.93 (9.79) | 0.87 (0.08, 1.66) ^*^ | 0.77 (0.02, 1.52) ^*^ | 0.79 (0.05, 1.54) |
| Quintile 3 | 70.20 (9.65) | 0.13 (-0.66, 0.93) | -0.09 (-0.85, 0.67) | -0.18 (-0.94, 0.57) |
| Quintile 4 | 70.62 (9.54) | 0.56 (-0.26, 1.38) | 0.31 (-0.48, 1.09) | 0.26 (-0.52, 1.04) |
| Quintile 5  (highest adherence) | 71.06 (9.51) | 0.99 (0.17, 1.82) ^*^ | 0.69 (-0.12, 1.49) | 0.55 (-0.25, 1.35) |
| Per 10-point increment | 70.56 (9.61) | 0.25 (0.02, 0.48) ^*^ | 0.15 (-0.07, 0.37) | 0.11 (-0.11, 0.33) |
| P for trend | - | 0.080 | 0.341 | 0.560 |
| **Women** | | | | |
| Quintile 1  (lowest adherence) | 66.10 (9.95) | 0.00 | 0.00 | 0.00 |
| Quintile 2 | 64.80 (9.47) | -1.29 (-1.81, -0.77) ^***^ | -0.72 (-1.19, -0.25) ^**^ | -0.71 (-1.18, -0.24) ^**^ |
| Quintile 3 | 64.72 (9.32) | -1.38 (-1.90, -0.86) ^***^ | -0.60 (-1.07, -0.12) ^*^ | -0.57 (-1.04, -0.10) ^*^ |
| Quintile 4 | 64.46 (9.33) | -1.63 (-2.15, -1.12) ^***^ | -0.63 (-1.10, -0.15) ^*^ | -0.66 (-1.13, -0.19) ^**^ |
| Quintile 5  (highest adherence) | 64.42 (9.16) | -1.68 (-2.19, -1.17) ^***^ | -0.55 (-1.02, -0.07) ^*^ | -0.57 (-1.04, -0.10) ^*^ |
| Per 10-point increment | 64.89 (9.46) | -0.43 (-0.57, -0.29) ^***^ | -0.09 (-0.23, 0.04) | -0.10 (-0.24, 0.03) |
| P for trend | - | <0.001 | 0.077 | 0.053 |

SD=standard deviation, CI=confidence interval.

P for interaction between sex and planetary health diet scores on heart age was lower than 0.001.

Model 1: adjusted for sex (for all participants only), education, occupation, family income, alcohol use, physical activity, self-rated health and objective health status.

Model 2: additionally adjusted for body mass index.

^*^P<0.05, ^**^P<0.01, ^***^P<0.001.

30,430 participants from GBCS Phase 1-3 (2003-2008)

29,089 participants with PHD score information remained

1426 participants with missing or unreliable energy intake, or with missing information on PHD scores

2607 participants with baseline CVD and 499 with baseline cancer

348 participants loss to follow-up for vital status

25,550 participants from GBCS Phase 1-3 (2003-2008) were included in the main analysis

**Supplementary Figure 1** **Flow chart of the study sample selection in the main analysis in Guangzhou Biobank Cohort Study (GBCS)**

GBCS=Guangzhou Biobank Cohort Study, CVD=cardiovascular disease, PHD=planetary health diet.


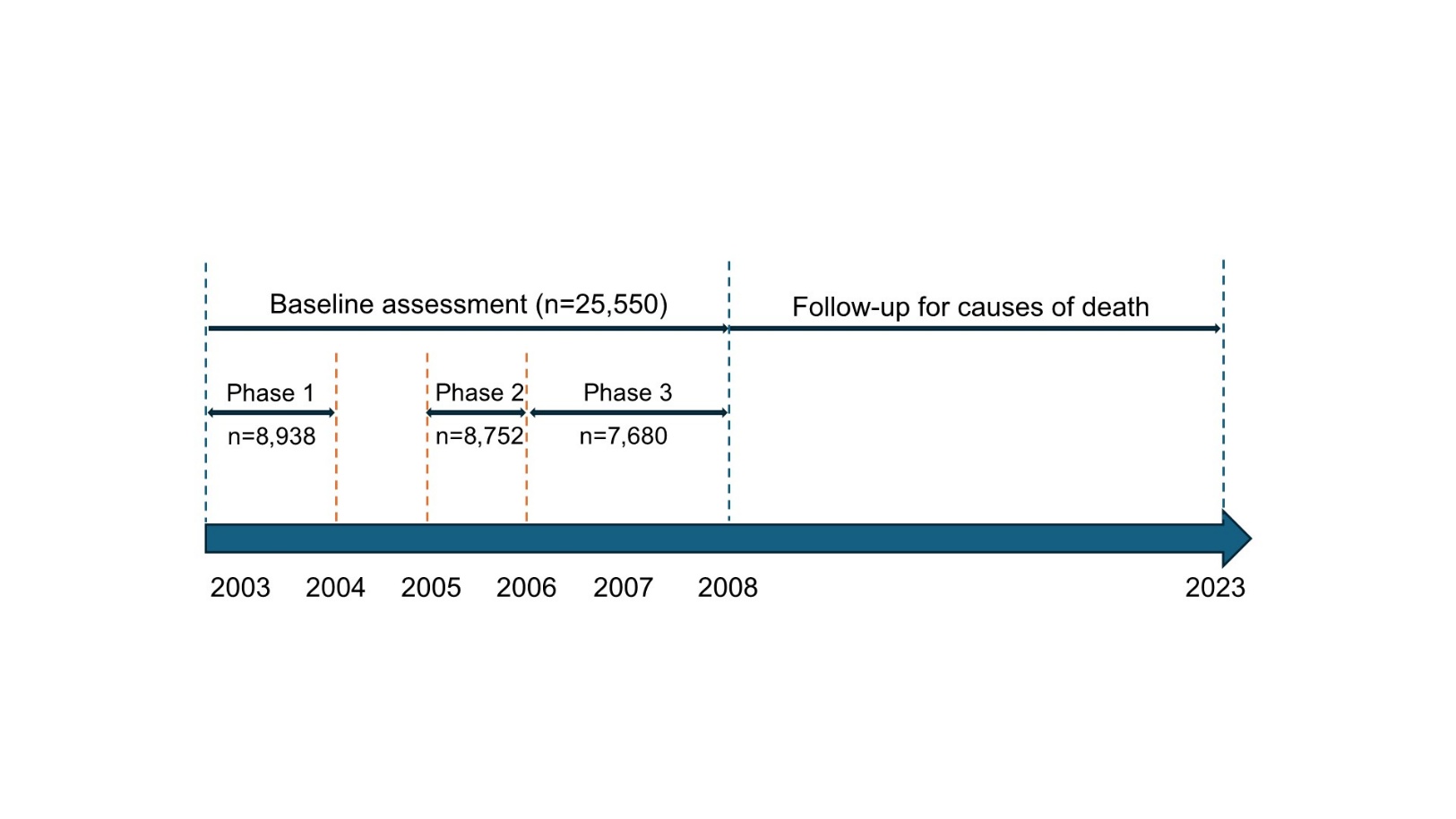


**Supplementary Figure 2 Timeline and the number of participants in the main analysis in Guangzhou Biobank Cohort Study**


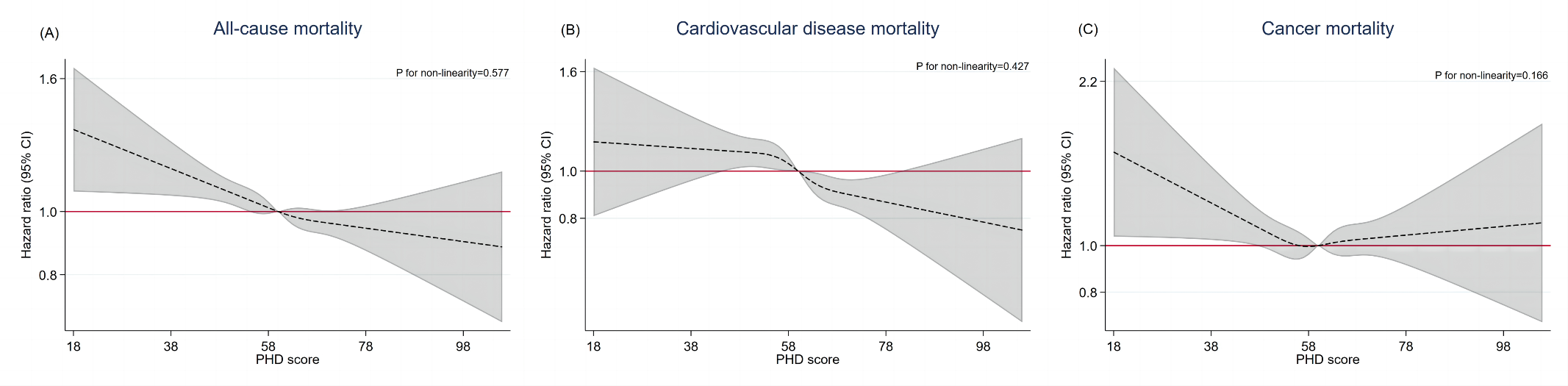


**Supplementary Figure 3 Restricted cubic spline plots for the associations of baseline planetary health diet scores with all-cause, cardiovascular disease and cancer mortality on 17,690 Guangzhou Biobank Cohort Study (GBCS) participants in 2003-2006 and followed up till November 2023**

HR=hazard ratio, CI=confidence interval, PHD=planetary health diet.

(A) all-cause mortality; (B) cardiovascular disease mortality; (C) cancer mortality. The HRs and 95% CIs above were adjusted for sex, age, education, occupation, family income, smoking status, alcohol use, physical activity, self-rated health, objective health status and body mass index.
